# Human Mesenchymal Stem Cell-Derived Extracellular Vesicles Promote Neural Differentiation of Neural Progenitor Cells

**DOI:** 10.3390/ijms23137047

**Published:** 2022-06-24

**Authors:** So-Yeon Park, Da-Seul Kim, Hyun-Mun Kim, Jun-Kyu Lee, Dong-Youn Hwang, Tae-Hyung Kim, Seungkwon You, Dong Keun Han

**Affiliations:** 1 Department of Biomedical Science, CHA University, 335 Pangyo-ro, Bundang-gu, Seongnam-si 13488, Korea; parks1201@korea.ac.kr (S.-Y.P.); dptmf4011@cau.ac.kr (D.-S.K.); kyn0708@naver.com (H.-M.K.); jklee2020@chauniv.ac.kr (J.-K.L.); hdy@cha.ac.kr (D.-Y.H.); 2 Division of Biotechnology, College of Life Sciences and Biotechnology, Korea University, Seoul 02841, Korea; 3 School of Integrative Engineering, Chung-Ang University, 84 Heukseok-ro, Seoul 06974, Korea; thkim0512@cau.ac.kr

**Keywords:** extracellular vesicle (EV), mesenchymal stem cell, neural progenitor cell, neurogenesis, microRNA, cytokine

## Abstract

Mesenchymal stem cells (MSCs) have been adopted in various preclinical and clinical studies because of their multipotency and low immunogenicity. However, numerous obstacles relating to safety issues remain. Therefore, MSC-derived extracellular vesicles (EVs) have been recently employed. EVs are nano-sized endoplasmic reticulum particles generated and released in cells that have similar biological functions to their origin cells. EVs act as cargo for bioactive molecules such as proteins and genetic materials and facilitate tissue regeneration. EVs obtained from adipose-derived MSC (ADMSC) also have neuroprotective and neurogenesis effects. On the basis of the versatile effects of EVs, we aimed to enhance the neural differentiation ability of ADMSC-derived EVs by elucidating the neurogenic-differentiation process. ADMSC-derived EVs isolated from neurogenesis conditioned media (differentiated EVs, dEVs) increased neurogenic ability by altering innate microRNA expression and cytokine composition. Consequently, dEVs promoted neuronal differentiation of neural progenitor cells in vitro, suggesting that dEVs are a prospective candidate for EV-based neurological disorder regeneration therapy.

## 1. Introduction

Mesenchymal stem cells (MSCs) have been widely used for decades in innumerable tissue engineering studies as candidates for cell-based therapy owing to their outstanding properties, such as their multipotency and immunomodulatory effects [1,2,3,4,5]. Specifically, stem cell therapy has been used to restore neurological disorders, including stroke, Alzheimer′s disease, and Parkinson’s disease. Urrutia et al. reported that adipose-derived MSCs (ADMSCs) exhibited stronger neural differentiation capacity than skin-derived MSCs, bone marrow-derived MSCs (BMMSCs), and umbilical cord-derived MSCs (UCMSCs) [6]. Recent studies have demonstrated that transplanted stem cells can differentiate into neuronal cells and glial cells and that substantial functional recovery is mostly due to paracrine signaling effects provided by grafted stem cells [7,8,9]. However, several safety issues, including immunogenic risk, still remain [10].

The extracellular vesicle (EV) is a crucial paracrine molecule of 30–150 nm in size with a bilayer structure. EVs exhibit tetraspanins CD63, CD81, and CD9 on the surface and present the endo-lysosomal regulatory transport protein (ALIX) [11,12]. MSC-derived EVs have been regarded to have a similar therapeutic potency as MSCs without the potential safety risks [10]. For the regeneration of neurological disorders, EV-based therapy is also promising as it is a neuroprotective paracrine cargo containing abundant proteins and microRNAs [13]. Therefore, Koohsari et al. studied the immunomodulatory effects of MSC-EVs alleviated by human UCMSC-induced experimental autoimmune encephalomyelitis (EAE) by reducing pro-inflammatory cytokine expressions (IL-17, TNF-α, and IFN-γ) and enhancing anti-inflammatory cytokine expression (IL-4 and IL-10) [14].

MicroRNA (miRNA), which are approximately 21 nucleotides long, are single-stranded RNA molecules originating from 70 to 100 nucleotide hairpin precursors that can regulate protein expression in recipient cells by binding to an untranslated mRNA region [15,16,17]. Previous studies validate the regenerative ability of miRNA, particularly their neurogenic properties [13,18]. For example, Kuang et al. reported that ADMSC-derived EVs containing miR-25-3p enhanced neuroprotective effects in a mouse stroke model by reducing the autophagy flux [11]. Upadhya et al. verified the potential therapeutic, anti-inflammatory and neurogenic properties of eight miRNAs (miR 320a, 320b, 103a-3p, 21-5p, 26a-5p, 30a-3p, 181a-5p, and 191-5p). A well-known miRNA capable of regulating neuronal differentiation is miR Let-7b, which inhibits neural stem-cell proliferation and promotes neural differentiation [19]. Further, miR 9 and miR 124 are extensively enriched in the brain and their expression levels can increase throughout the mature nervous system. The miR-9 controls the post-differentiation regulation of embryonic and adult neural stem cells [20]. Existing miR 124 can be a transcriptional repressor of the RE1-silencing transcription factor that downregulates the expression of neuronal genes in nonneuronal cells [21]. Hence, overexpressed Let-7b, miR 9, and miR 124 in EVs can effectively enhance the neuronal differentiation of recipient cells.

Moreover, new EV isolation methods have been developed to enhance EV regenerative properties using diverse biophysiological stimulation. For example, in bone regeneration studies, several studies showed that EV derived from osteogenic differentiation stages increased the osteogenic effects of EV [22,23].

In this study, we hypothesized that EVs isolated from neurogenesis-conditioned media can increase the neurogenic ability of EVs (differentiated EVs: dEVs) compared with normal ADMSC-derived EVs. Additionally, we previously compared innate characteristics of EVs based on cell culture media, namely the serum containing DMEM/F12 media and serum-free chemically defined media (CDM). CDM has the inherent advantage of enhancing cell proliferation and eliminating impurities during EV isolation [10]. A comparative analysis of EVs isolated from neurogenesis conditioned with serum-supplemented DMEM/F12 media (dEVs-F12) and CDM (dEVs-CDM) suggests a new EV-based therapeutic approach for neurogenic diseases.

## 2. Results and Discussion

### 2.1. dEVs Isolation during Neuronal Differentiation of ADMSCs

**Figure 1:**
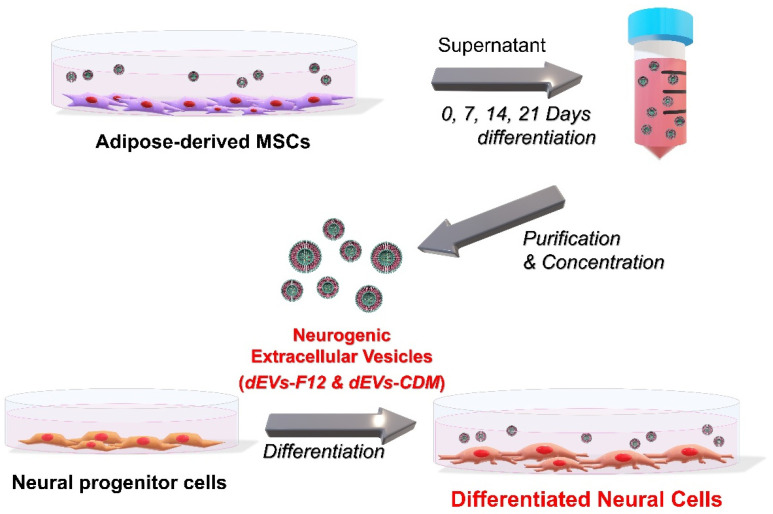
Schematic illustration of dEV isolation. For the effects of dEVs on neuronal differentiation of neural progenitor cells (NPCs), ADMSC-derived supernatant media were collected under neural differentiation conditions at 0, 7, 14, and 21 days.

During ADMSC neurogenesis, EV properties can change due to altered cell–cell interactions. Therefore, we hypothesized that EVs secreted from neural-differentiated ADMSCs show neurogenic abilities. To isolate dEVs, FBS-derived redundant EVs were eliminated in DMEM/F12 media containing serum following a previous study [10]. Cultured media were collected from ADMSCs under neural differentiation conditions at 0, 7, 14, and 21 days (Figure 1). Subsequently, the supernatant was isolated using a tangential flow filtration (TFF) system.

**Figure 2:**
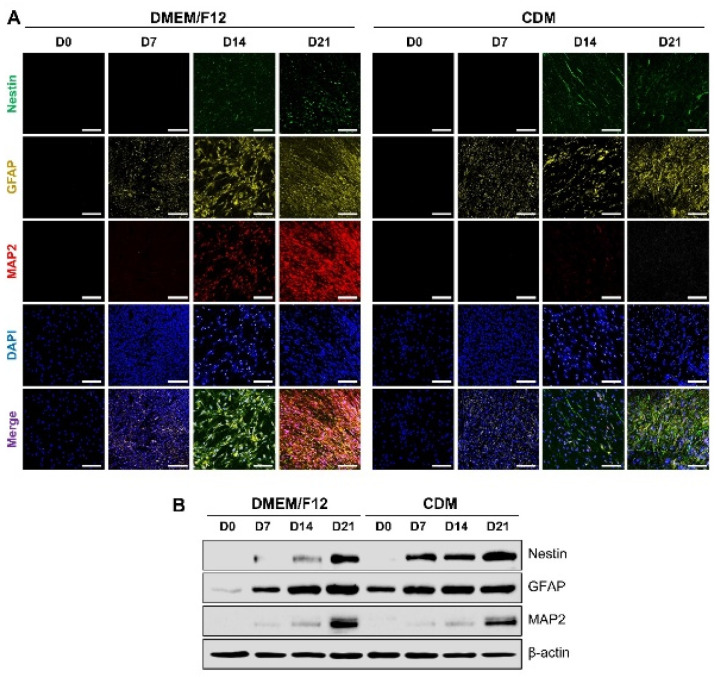
Confirmation of neuronal differentiation ability of ADMSCs. (**A**) Representative immunofluorescence staining images of differentiated ADMSCs labeled with neuronal markers: Nestin (green), GFAP (yellow), and MAP2 (red) after 0, 7, 14, and 21 days under neural differentiation conditions. DAPI (blue) represents the nuclei of ADMSCs. Scale bars indicate 200 μm. (**B**) Western blot analysis results with neuronal markers: Nestin, GFAP, and MAP2. β-actin served as a loading control.

First, to investigate the differentiation behavior of multipotent ADMSCs in neurogenesis media, which are the conditions required for EV isolation, immunocytochemistry (ICC) analysis was conducted for staining of neuronal cell-specific proteins (Figure 2A). Differentiated ADMSCs were visualized with Nestin as a neural progenitor cell marker, glial fibrillary acidic protein (GFAP) (an intermediate filament) as an astrocyte marker, and microtubule-associated protein 2 (MAP2) as a mature neuron marker [24,25,26,27,28,29].

After 14 days of differentiation, Nestin and GFAP expression were observed in both DMEM/F12 and CDM media groups. Interestingly, in the DMEM/F12 group, MAP2 was also expressed. For DMEM/F12 D21, we explored the highest MAP2 expression. Similarly, neuronal cell-related protein expressions were investigated using Western blot analysis (Figure 2B). Further, MAP2 expression of differentiated ADMSCs increased in the DMEM/F12 D21 group more than in the CDM D21 group.

Based on the differentiation results from ADMSCs, the neurogenesis of ADMSC with DMEM/F12 was enhanced for differentiation into mature neurons. However, CDM-based differentiation showed a tendency to dominate differentiated astrocyte-positive cells instead of neurons.

### 2.2. Characterization of Neuronal Differentiated ADMSC-Derived dEVs

**Figure 3:**
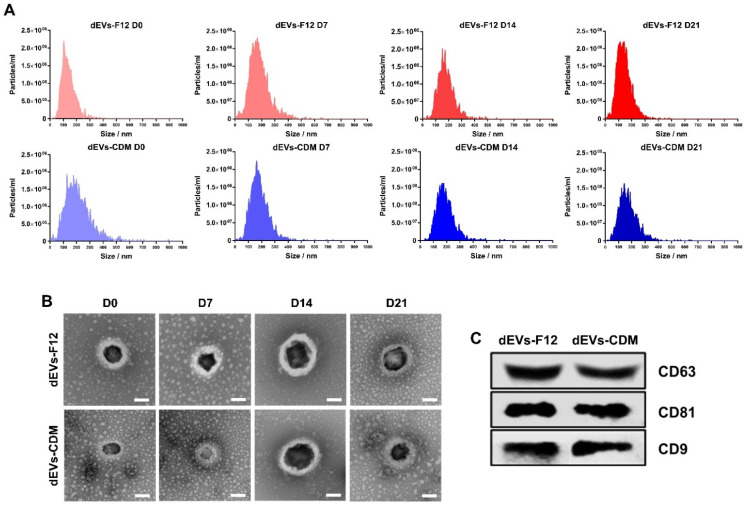
Characterization of neuronal differentiated ADMSC-derived dEVs. (**A**) Confirmation of the average number and size distributions of dEVs using Zetaview. (**B**) Representative images of transmission electron microscopy (TEM) of isolated dEVs. Scale bars indicate 100 nm. (**C**) Western blot analysis showing expression of EV-specific surface markers (CD63, CD81, and CD9) in dEVs.

EV-specific protein expression and EV morphology were demonstrated following the guidelines of MISEV2018 to verify the characteristics of dEVs-F12 and dEVs-CDM [30]. Based on nanoparticle tracking analysis (NTA) results using Zetaview QUATT^®^, the size distributions of dEVs showed heterogeneity within a widely accepted range (diameter 50–300 nm) given the complexity of varying sizes and compositions [8,31]. There was no significant difference in particle numbers between dEVs-F12 and dEVs-CDM media groups. Average particle numbers were 2.1 × 10^10^ for dEVs-F12 and 1.97 × 10^10^ for dEVs-CDM (Figure 3A).

The bilayered spherical structure of dEVs was confirmed using transmission electron microscopy (TEM) to determine whether the shape was maintained following separation and concentration by TFF. In addition, average dEVs-F12 size was 215, 214, 249, and 223 nm (D0, 7, 14, and 21, respectively) based on TEM images (Figure 3B). Similarly, average dEVs-CDM sizes were 149, 173, 261, and 215 nm (D0, 7, 14, and 21, respectively).

Tetraspanin molecules, including CD63, CD81, and CD9, are the most common surface protein markers of EVs [32] and play an essential role in numerous biological processes for cell–cell interactions, such as cell adhesion, membrane fusion, signal transduction, and protein trafficking [32]. We performed Western blot analysis to determine the expression of surface markers on dEVs (Figure 3C) and demonstrated that there were no relevant changes in neither production yield nor intrinsic surface properties of ADMSC-derived EVs, dEVs-F12, or dEVs-CDM during neuronal differentiation.

### 2.3. Enhancing Neurogenesis of NPCs Using dEVs

**Figure 4:**
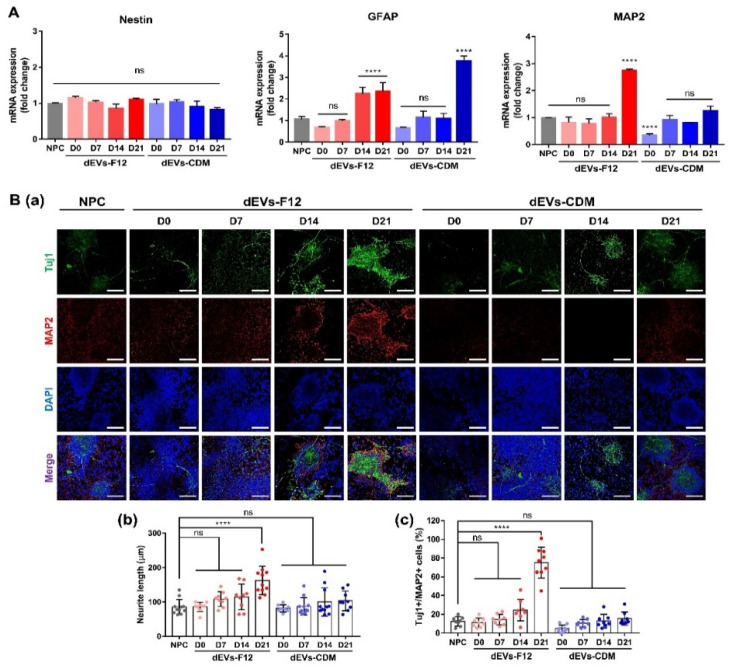
Enhanced neuronal differentiation effects of dEVs using NPCs under neural differentiation conditions after 7 days. (**A**) Neurogenesis-related gene expressions of dEV treated NPCs using RT-qPCR. (**B**) Representative images of immunofluorescence staining of differentiated NPCs labeled with neuronal markers (**a**) Tuj1 (green) and MAP2 (red). DAPI (blue) represents the nuclei of NPCs. Scale bars indicate 100 μm. Quantifying neurite length (**b**) and Tuj-1/MAP2 copositive cell numbers (**c**). **** *p* < 0.0001 and ns = no significant. One-way ANOVA was followed by Tukey’s test. Error bars represent standard deviation (SD).

NPCs are characterized by their ability to expand and generate the majority of differentiated cell types in the central nervous system: neurons, astrocytes, and oligodendrocytes under specific environments [33]. Therefore, the differentiation ability of dEVs was analyzed using neurogenesis-induced NPCs. Quantitative reverse transcription-polymerase chain reaction (RT-qPCR) was performed on NPCs differentiated for 7 days with each dEV treatment (Figure 4A). Nestin mRNA expression levels in dEVs exhibited negligible differences compared with controls. The dEV-treated NPC group notably increased GFAP expression by approximately 1.24–1.35-fold in dEVs-F12 D14 and D21 compared with controls. Further, GFAP expression of dEVs-CDM D21 treated NPCs was significantly upregulated by 2.76-fold compared with controls (*p* < 0.0001). Most importantly, the expression of the mature neuron marker MAP2 was remarkably accelerated in dEVs-F12 D21 treated NPCs (2.73-fold more than controls, *p* < 0.0001).

To further investigate the neurogenesis capacity of dEV, we assessed neuronal skeletal markers (neuron-specific class III beta-tubulin, Tuj1) and mature neuron markers (MAP2) using ICC analysis. In Figure 4(Ba), based on representative images of differentiated NPCs, the dEVs-F12 D21 group demonstrated outstanding promotional effects on neuronal differentiation. Further, the length of neurite labeled with Tuj1 indicates elongated axons and multiple neurite outgrowth. The dEVs-F12 D21 group displayed the most significant elongation effect on neurite length following an 89.8% increase compared with the NPC. Figure 4(Bc) shows a differentiated NPCs population, stained double-positive for Tuj1 and MAP2, displayed the largest in the dEVs-F12 D21 group (607% higher than controls). In other words, dEVs-F12 D21 encouraged NPC neurogenesis not only for normal ADMSC-derived EV (dEVs-F12 D0), but also for other dEVs.

### 2.4. dEVs-F12 Promotes Neuronal Differentiation of NPCs by Altering Innate miRNA and Protein Composition

The paracrine effect of EVs on cells occurs via the transport of genetic material, mRNA, or miRNA [34,35]. As mentioned above, miRNAs (Let-7b, miR 9, and miR 124) are single-stranded noncoding RNAs expressed during the neuronal differentiation process [20,21]. Specifically, Let-7b and miR 9 inhibit the proliferation of neural stem cells and enhance the neuronal differentiation ability of NPCs by targeting the stem cell regulators TLX and TRIP6 and the cell cycle regulator cyclin D1 [36,37]. Furthermore, Kapsimali et al. described that in mammalian neurons, miR 124 induces neurogenesis and presents widespread in most CNS neurons [38].

**Figure 5:**
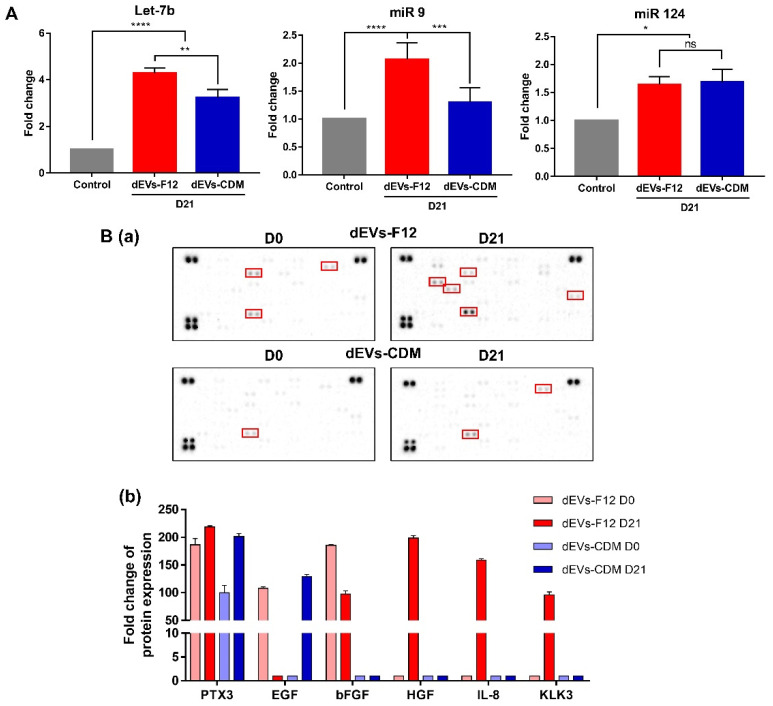
miRNA expressions for Let-7b, miR 9, and miR 124. (**A**) Quantification of the miRNA expressions related to neurogenesis of dEVs using RT-qPCR. (**B**) Cytokine expressions in dEVs. Representative images of cytokine array membranes (**a**) and quantification as a fold change (**b**). **** *p* < 0.0001, *** *p* < 0.001, ** *p* < 0.01, * *p* < 0.05, and ns = no significant. One-way ANOVA was followed by Tukey’s test. Error bars represent standard deviation (SD).

Therefore, to demonstrate changes inside dEV molecules, miRNA expression levels related to neural differentiation were evaluated [39]. RT-qPCR was performed to determine miRNA expression in dEVs (Figure 5A). Expression of miR 9 increased 2-fold in dEVs-F12 and 1.4-fold in dEVs-CDM compared with controls. Let-7b expressions elevated 4.31-fold in dEVs-F12 and 3.24-fold in dEVs-CDM. Further, miR 124 levels in both dEVs-F12 and dEVs-CDM upregulated 1.6-fold. All miRNAs were compared with control-differentiated NPCs.

To further assess the paracrine effects of dEVs, EV lysate was characterized using a cytokine array (Figure 5B). Cytokine arrays were executed to investigate proteins that functioned in NPC differentiation along with miRNAs. Cytokines highly expressed in dEVs-F12 included pentraxin 3 (PTX3), fibroblast growth factor-Basic (bFGF), hepatocyte growth factor (HGF), interleukin 8 (IL-8), and Kallikrein 3 (KLK3) that were markedly expressed to 218-, 98-, 198-, 159-, and 96-fold compared with controls, respectively. All cytokines can improve neuron survival and proliferation. Specifically, PTX3, an activator of the A2 astrocytes, mediates neurogenesis and helps restore nerve function via angiogenesis [40,41]. bFGF supports the growth of nerve cells and neural stem cells and can protect nerves [42], whereas HGF can prevent neuronal cell death [43]. Furthermore, KLK3 can promote neurite outgrowth [44], and IL-8 increases neuronal survival [45]. Taken together, dEVs-F12 D21 enhanced the differentiation of NPCs by changing internal bioactive molecules related to neurogenesis.

## 3. Materials and Methods

### 3.1. Neuronal Differentiation of ADMSCs into Neural Lineage Cells

The ADMSCs (passage 5, CEFOBIO, Seoul, Korea) were confirmed by flow cytometry (CytoFLEX; Beckman Coulter, CA, USA). The representative surface expression markers of hMSCs, CD73(130-097-943, BD Biosciences, New Jersey, USA) and CD105 (562408, BD Biosciences, New Jersey, USA), and the non-expression marker, CD34 (555822, BD Biosciences, New Jersey, USA) were used for analysis. ADMSCs were cultured in 150 mm TC-treated culture dishes (430599, Corning, New York, USA) with a density of 1 × 10^6^ cells/mL. ADMSCs were cultured in DMEM, low glucose (LM00111, Welgene, Gyeongsangbuk-do, Korea), 100× Antibiotic-Antimycotic 1% (AA, 15240062, Gibco, New York, USA), and Premium Fetal Bovine Serum 5% (FBS, 35015CV, Corning, New York, USA) to reach 60% confluency, then neural differentiation was induced by two types of media, existing DMEM/F-12, no phenol red (21041025, Gibco, New York, USA) and a new type of CellCor™ CD MSC (CDM, XCELL Therapeutics, Seoul, Korea). Both media contained N-2 Supplement 1× (17502048, Gibco, New York, USA), B-27 Supplement 1× (12587010, Gibco, New York, USA), Recombinant Human FGF-basic 20 ng/mL (100-18b, Peprotech, New Jersey, USA), Animal-Free Recombinant Human EGF 20 ng/mL (AF-100-15, Peprotech, New Jersey, USA), AA 1% (15240062, Gibco, New York, USA), and retinoic acid 1 μM (R2625, Sigma Aldrich, MI, USA). Additionally, DMEM/F-12, no phenol red medium contains 5% ultracentrifugation FBS. The medium was changed every 2–3 days.

### 3.2. Neuronal Differentiation of NPCs

Neural progenitor cells (NPCs) were cultured with N-2 Supplement 1× (Thermo Fisher Scientific, Massachusetts, USA), B-27 Supplement 1× (Thermo Fisher Scientific, Massachusetts, USA), epidermal growth factor (EGF, 20 ng/mL, Peprotech, New Jersey, USA), basic fibroblast growth factor (bFGF, 20 ng/mL, Peprotech, New Jersey, USA) in DMEM/F12 media. NPCs were seeded in 24-well cell culture plates coated with Matrigel (354234, 1/200, Corning, New York, USA). Cells were cultured in an incubator at 5% CO₂ and 37 °C. Cells were subcultured every 4–5 days, and the medium was changed daily.

For neuronal differentiation, NPCs were changed with DMEM/F12 medium containing N-2 Supplement 1×, B-27 Supplement 1×, brain-derived neurotrophic factor (BDNF, 10 ng/mL, Peprotech, New Jersey, USA), glial cell-derived neurotrophic factor (GDNF), 10 ng/mL, Peprotech, New Jersey, USA), nerve growth factor (NGF, 10 ng/mL, Peprotech, New Jersey, USA), and 0.1 mM L-Ascorbic acid (Sigma Aldrich, Missouri, USA) with 5 × 10^7^ particles/mL of dEVs. The medium was changed daily, and the cells were cultured for 7 days.

### 3.3. Characterization of Differentiation ADMSC-EVs

After 0, 7, 14, and 21 days of neuronal differentiation, dEVs were isolated from ADMSCs cultured media for every 48 h. The dEVs were concentrated using the tangential flow filtration (TFF, Repligen, Massachusetts, USA) system with a 500 kDa fiber membrane (KR2i TFF system, Repligen, Massachusetts, USA), and the isolated dEVs were concentrated with Amicon Ultra-2 10 K (Merck Millipore, Massachusetts, USA) as previously described [46]. The morphology of the dEVs was observed with transmission electron microscopy (TEM, H-7600, Hitachi, Japan). For TEM analysis, the EV solution was diluted and dropped on the copper grid with 200 mesh carbon support film (CF200-CU, Electron Microscopy Sciences, Pennsylvania, USA). Representative TEM images of dEV were obtained using a FEI Tecnai Spirit G2 (FEI Company, Oregon, USA). The particle concentration and size distribution were analyzed by nanoparticle tracking analysis (NTA) using a Nanosight NS300 (Malvern Instruments, Worcestershire, UK) and Zetaview QUATT (Particle Metrix, Inning am Ammersee, Germany) was used to identify the number of EV particles with scatter mode. The instrument settings were set to a sensitivity of 75 and a shutter speed of 100 for all samples.

### 3.4. Immunocytochemistry

Cells were fixed in 4% paraformaldehyde (Biosesang, Korea) for 15 min at room temperature (RT) and permeabilized for 15 min with 0.2% Triton X-100 (Sigma-Aldrich, Missouri, USA) in phosphate-buffered saline (PBS) solution, followed by being blocked with diluted 1% ready probe normal horse serum (R37625, Invitrogen, Massachusetts, USA) in 1% BSA solution. After cells were incubated with primary antibodies; Anti-MAP2 antibody (ab5392, 1:500, Abcam, Cambridge, UK), Recombinant anti-Nestin antibody (ab105389, 1:200, Abcam, Cambridge, UK), Anti-beta III Tubulin antibody (ab18207, 1:500, Abcam, Cambridge, UK), and mouse anti-GFAP (556327, 1:500, BD Biosciences, New Jersey, USA) at 4 °C overnight. The next day, cells were stained with secondary antibodies in serum-BSA for 1 h at RT and incubated with 1 μg/mL of Hoechst dye (33342, Invitrogen, Massachusetts, USA) for 15 min at RT. Cells were washed three times with PBS solution in all steps. Confocal images were acquired using a confocal laser microscope (LSM880, Carl Zeiss, Germany). To quantify the neurite length and Tuj1/MAP2 copositive cells, ImageJ software was utilized.

### 3.5. Western Blot Analysis

dEVs and differentiated ADMSCs were incubated with radioimmunoprecipitation assay (RIPA) Buffer 1× (9806S, Cell Signaling, Massachusetts, USA) for 30 min, and centrifuged for 10 min. Proteins were measured by The Pierce^TM^ BCA Protein Assay Kit (Pierce, Illinois, USA). dEVs were separated on 10% SDS-PAGE gel and cell proteins were separated on 8%. Subsequently, the gels were transferred onto nitrocellulose membranes. The membranes were blocked with 5% skim milk dissolved Tris-buffered saline with Tween (TBS-T) solution for 1 h at RT. Each membrane was sequentially incubated with the primary antibody of Anti-CD9 antibody (1:200, Santa Cruz Biotechnology, Texas, USA), Anti-CD63 antibody (1:500, Abcam, Cambridge, UK), Anti-CD81 (1:200, Santa Cruz Biotechnology, Texas, USA), Anti-MAP2 antibody (ab5392, 1:500, Abcam, Cambridge, UK), Recombinant Anti-Nestin Antibody (ab105389, 1:200, Abcam, Cambridge, UK), and Mouse anti-GFAP (556327, 1:500, BD Biosciences, New Jersey, USA) at 4 °C overnight. The next day, anti-Mouse IgG, HRP-linked secondary antibody (1:5000, Cell Signaling, Massachusetts, USA) and anti-Rabbit IgG, HRP-linked Antibody (1:5000, Cell Signaling, Massachusetts, USA) were incubated for 1 h. The expressions were detected using Chemi Doc^TM^ XRS and Image Lab software (Bio-Rad, California, USA).

### 3.6. RT-qPCR Analysis

Differentiated ADMSCs and NPCs were isolated using an AccuPrep Universal RNA Extraction Kit (Bioneer, Gyeonggi-do, Korea). The Prime Script RT Reagent Kit (Takara Biotechnology, Shiga, Japan) was used for complementary DNA (cDNA) synthesis and quantitative reverse transcription PCR (RT-qPCR) process. RT-qPCR was performed using a Power SYBR green PCR master mix (Applied Biosystems, Massachusetts, USA). All data were normalized against the 18 s rRNA. Forward and reverse primer sequences were designed for Nestin, GFAP, MAP2, and 18S rRNA as follows: Nestin (forward: 5′-TGCGGGCTACTGAAAAGTTC-3′; reverse: 5′-AGGCTGAGGGACATCTTGAG-3′), GFAP (forward: 5′-CAGGTCCATGTGGAGCTTGAC-3′; reverse: 5′-CATTGCCTCATACTGCGTGC-3′), MAP2 (forward: 5′-TGTACCTGGAGGTGGTAATGTC-3′; reverse: 5′-TGGCAAGCTGAGGAGATTCGAGC-3′), and 18S (forward: 5′-CCTGGATACCGCAGCTAGGA-3′; reverse: 5′-GCGGCGCAATACGAATGCCCC-3′). miRNAs were extracted from dEVs by the miRNeasy Kits (Qiagen, Shanghai, China). For the analysis of miRNAs (mir 9, 124, 137-3, and 138, let-7b) expression, based on the manufacturer’s instructions, Mir-X miRNA RT-qPCR SYBR Kit (Clontech, California, USA) was used for cDNA synthesis and RT-qPCR process. U6 snRNA was used as a control for quantification. All data were calculated by the 2^−ΔΔCt^ method and obtained on a QuantStudio3 real-time PCR system (Applied Biosystems, Massachusetts, USA). Primer sequences were designed for hsa-miR-9-5p, hsa-miR-124-3p, and let-7b as follows: hsa-miR-9-5p (5′-TCTTTGGTTATCTAGCTGTATG-3′), hsa-miR-124-3p (5′-TAAGGCACGCGGTGAATGCCAA-3′), and let-7b (5′-TGAGGTAGTAGGTTGTGTGGTT-3′).

### 3.7. Cytokine Array

dEVs were lysed in RIPA Buffer 1× (9806S, Cell Signaling, Massachusetts, USA) and the 100 μg of dEVs protein was analyzed by a Proteome Profiler Human XL Cytokine Array Kit (ARY022B, R&D Systems, Minnesota, USA) according to the manufacturer’s protocol. Each cytokine was revealed in duplicate on the array plate, and the average of spots was taken for the analysis of each factor expression. The fold change of cytokine productions was calculated over the background using densitometry as measured by ImageJ software.

### 3.8. Statistical Analysis

All experiments were repeated at least three times. The quantitative results were presented as means ± standard deviation (SD). # *p* < 0.0001, *** *p* < 0.001, ** *p* < 0.01, and * *p* < 0.05 indicate statistically difference, respectively. Statistically significant differences were evaluated by one-way analysis of variance (ANOVA) following Tukey’s method in GraphPad Prism 7.0 software (GraphPad Software Inc., California, USA).

## 4. Conclusions

In the field of tissue engineering and regenerative medicine, particularly with respect to neurodegenerative diseases, numerous approaches have been studied to develop effective cell-free therapeutic methods using MSC-derived EVs [1,11,13,47]. As mentioned previously, to eliminate the effect of FBS-derived EVs and enhance the inherent effects of EVs in cells of interest, we applied serum-free CDM. Although CDM has several advantages, such as maintaining cell proliferation, enhancing angiogenesis, and reducing impurities for EV isolation [10], DMEM/F12 still showed promising results in neuronal differentiation of ADMSCs. In future studies, component modification of CDM for neuronal differentiation will leverage these results.

However, ultimately, dEVs-F12 promotes neuronal differentiation of NPCs by altering the innate miRNA composition (Let-7b, miR 9, and miR 124) in EVs. Furthermore, inherent cytokines in dEVs-F12 promote neurogenesis, protect neurons, and enhance proliferation. Our findings offer insight into the future therapeutic use of EVs for neuronal disorders in the fields of tissue engineering and regenerative medicine.

## Data Availability

Required data files will be available upon request.

## Abbreviations

NPCs	Neural progenitor cell
ADMSC	Adipose-derived mesenchymal stem cells
CDM	Chemically defined media
TFF	Tangential flow filtration
dEV	Neuronal differentiated ADMSC-derived extracellular vesicle (Differentiated EV)
NTA	Nanoparticle tracking analysis
miRNA	microRNA
GFAP	Glial fibrillary acidic protein
Tuj1	Tubulin beta III
MAP2	Microtubule-associated protein 2
PTX3	Pentraxin 3
bFGF	Fibroblast growth factor-Basic
HGF	Hepatocyte growth factor
IL-8	Interleukin 8
KLK3	Kallikrein 3
